# Increased primary care use for musculoskeletal symptoms, infections and comorbidities in the years before the diagnosis of inflammatory arthritis

**DOI:** 10.1136/rmdopen-2019-001163

**Published:** 2020-07-20

**Authors:** Marian van Beers-Tas, Markus MJ Nielen, Jos W R Twisk, Joke Korevaar, D van Schaardenburg

**Affiliations:** 1 Rheumatology, Amsterdam Rheumatology and Immunology Center, Reade, Amsterdam, Netherlands; 2 Netherlands Institute for Health Services Research (NIVEL), Utrecht, Netherlands; 3 Department of Epidemiology and Biostatistics, VU University Medical Center, Amsterdam, Netherlands; 4 Rheumatology, Amsterdam Rheumatology & immunology Center, Amsterdam University Medical Center location AMC, Amsterdam, The Netherlands

**Keywords:** Autoantibodies, Rheumatoid Arthritis, Inflammation

## Abstract

**Objectives:**

Little is known about relevant events in the at-risk phase of rheumatoid arthritis before the development of clinically apparent inflammatory arthritis (IA). The present study assessed musculoskeletal symptoms, infections and comorbidity in future IA patients.

**Methods:**

In a nested case–control study using electronic health records of general practitioners, the frequency and timing of 192 symptoms or diseases were evaluated before a diagnosis of IA, using the International Classification of Primary Care coding system. Cases were 2314 adults with a new diagnosis IA between 2012 and 2016; controls were matched 1:2. The frequency of primary care visits was compared using logistic regression.

**Results:**

The frequency of visits for musculoskeletal symptoms (mostly of shoulders, wrists, fingers and knees) and carpal tunnel syndrome was significantly higher in IA patients vs controls within the final 1.5 years before diagnosis, with ORs of 3.2 (95% CI 2.8 to 3.5), 2.8 (95% CI 2.5 to 3.1) and 2.5 (95% CI 2.2 to 2.8) at 6, 12 and 18 months before diagnosis, respectively. Also, infections (notably of the genital and urinary tracts), IA-comorbidities and chronic diseases were more prevalent in cases than controls, but more evenly spread out over the whole 6-year period before IA. A decision tree was created including all symptoms and diseases.

**Conclusion:**

There was an increased frequency of primary care visits for musculoskeletal symptoms, infections and comorbidities prior to the diagnosis of IA. This diverging trend is present for 4–6 years, but becomes statistically significant 1.5 years before the diagnosis. Validation of these results is warranted.

## INTRODUCTION

Rheumatoid arthritis (RA) is usually diagnosed shortly after the appearance of clinically apparent inflammatory arthritis (IA). The time between the onset of persistent joint symptoms and the diagnosis RA by the rheumatologist varies^1^; in the Netherlands, the median duration is 4 months.^2,3^ Early recognition and start of treatment improve the outcome.^3^ General practitioners (GPs) can play an essential role in earlier detection of IA as they are the first professional to be consulted for health problems and all Dutch inhabitants are listed with a GP. Furthermore, the GP has a gatekeeper role and therefore refers a patient with suspected IA to the rheumatologist. GPs have a complete overview of all health problems in their electronic health records (EHRs). The unique healthcare system in the Netherlands makes it possible to study symptom and morbidity patterns before the diagnosis.

It appears that GPs mostly use classical signs of inflammation such as pain and swelling to identify those with a high probability of having IA, and that those signs are the triggers for referral to secondary care.^4^ However, additional symptoms or conditions may occur before the diagnosis that are at that time not attributed to emerging RA, but do lead to increased ambulatory care utilisation.^5^ This is underscored by a higher rate of sick leave already 8 months before the first prescription of antirheumatic drugs.^6^ Also, the number of comorbid diseases at the onset of IA is higher than in a control group, however, it is not clear whether these diseases were already present before the onset of IA.^7^

In the phase before clinical RA, subclinical autoimmunity and inflammation often occur for several years.^8,9^ This may be related to the influence of environmental factors, such as infections or lifestyle factors.^10^ However, little is known about symptoms, pathogenetic events, other diseases and their timing during this phase.^11^ Also, available clues mostly come from case–control studies and studies of at-risk populations. These studies have the limitation that only selected individuals are studied, usually after referral to secondary care because of more severe symptoms.^11,12^ This limitation can be obviated by studying the at-risk phase of RA in the unselected primary care setting.

The present study focuses on pre-existing symptoms and diseases that are possibly related to RA, with the goals to improve early identification of future IA patients and to identify possible pathogenetic clues. Data from EHRs of GPs from a large Dutch national database were used to answer the following research questions: (1) To what extent are musculoskeletal symptoms, infections and/or RA-related comorbidities more prevalent before the diagnosis IA compared to control patients?, (2) What is the lead time between these early symptoms or disorders and the diagnosis IA?, (3) Is it possible to identify a combination of symptoms and diseases that can be used to predict IA development?

## PATIENTS AND METHODS

### Study population

Data were used from Nivel Primary Care Database (Nivel-PCD).^13^ Nivel-PCD collects data from routine EHR systems from a representative sample of approximately 500 general practices with a total of more than 1.5 million registered patients, including information about consultations, morbidity, prescriptions and diagnostic tests. In the Netherlands, EHR systems have been used for many years, and among other things, a guideline exists to help GPs to uniformly record complete qualitative data.^14,15^ Diagnoses were recorded using the International Classification of Primary Care (ICPC-1) coding system.^16^ Only data with sufficient quality were used: GPs had to have recorded data at least 46 weeks of the year with at least 70% ICPC coded visits. Adult patients (≥18 years) were selected based on having a new diagnostic code of IA (ICPC code L88) in the years 2012 to 2016, where identifying only incident cases with at least 1 year (with a maximum of 6 years) retrospective follow-up. L88 includes RA, psoriatic arthritis and ankylosing spondylitis,^17^ which in the ICPC coding system cannot be coded individually. In case, the start date of IA was preceded by the prescription of a disease-modifying antirheumatic drug (DMARD) and/or biological, we assumed that documentation of the L88 code might have been delayed and the date of diagnosis was set on the start date of the first DMARD or biological. Selection included: methotrexate, leflunomide, sulfasalazine, abatacept, rituximab, etanercept, infliximab, adalimumab, certolizumab, golimumab, tocilizumab, anakinra and ustekinumab. Use of hydroxychloroquine was allowed before the diagnosis of IA since this is prescribed occasionally in the at-risk phase in patients not having arthritis. Each case was matched with two controls (without IA in the past) in the same general practice based on age (±3 years), gender and duration of follow-up (depending on the registration date of the patient in a general practice, and registration of that particular general practice in Nivel-PCD).

### Procedures

We used data from EHRs containing information on consultations and prescriptions before the IA-date or matched end date of the control patients in the period 2006 to 2016. Consultations are mostly physical visits of patients to the GP, but can also be consultations by telephone or a debrief from a secondary care specialist. Throughout the rest of the manuscript, the term primary care visits is used. Prescriptions are those started by the GP as well as repeat prescriptions of medication started in secondary care. We preselected a list of 192 ICPC codes (online supplementary table 1) deemed relevant to RA development, which included musculoskeletal symptoms, infectious diseases and RA-related comorbidities. This selection was based on biological plausibility, literature research^5,11,18^ and expert opinion. On the one hand, we included codes described earlier to be associated with RA (mostly RA-related comorbidities) or hypothesised to be related (such as cardiac problems related to other autoimmune diseases like ankylosing spondylitis) and, on the other hand, we included codes that may trigger GPs to think of the diagnosis of arthritis and be more aware of its presence (such as carpal tunnel syndrome, peripheral neuritis and musculoskeletal symptoms in general). Since it is assumed that infections may trigger the development of autoimmune phenomena, we included all ICPC codes addressing specific infections in different body parts. In Nivel-PCD comorbidities and chronic diseases are coded separately, as comorbidities can be diagnosed more than once and chronic diseases only once.

**Table 1:** Univariable logistic regression analysis of the relation of individual ICPC codes with IA development

ICPC	Description	Group	OR	CI	P value
L20	Joint symptom/complaint NOS	Musculoskeletal	8.1	5.8–11.3	<0.01
L97	Chronic internal derangement knee	Musculoskeletal	5.9	1.6–21.8	<0.01
L11	Wrist symptom/complaint	Musculoskeletal	4.9	3.2–7.5	<0.01
NA	Other infectious symtoms	Infections	4.9	1.5–15.7	<0.01
L12	Hand/finger symptom/complaint	Musculoskeletal	4.0	3.1–5.1	<0.01
S91	Psoriasis	Chronic disease	3.7	2.5–5.4	<0.01
D94	Chronic enteritis/ulcerative colitis	Chronic disease	3.5	1.9–6.4	<0.01
T92	Gout	Chronic disease	3.5	2.6–4.7	<0.01
L29	Symptom/complaint musculoskeletal other	Musculoskeletal	2.9	1.9–4.4	<0.01
N93	Carpal tunnel syndrome	Musculoskeletal	2.7	1.9–4.0	<0.01
L92	Shoulder syndrome	Musculoskeletal	2.6	2.0–3.4	<0.01
L91	Osteoarthrosis other	Chronic disease	2.6	1.9–3.5	<0.01
L19	Muscle symptom/complaint NOS	Musculoskeletal	2.5	1.6–4.0	<0.01
B80	Iron deficiency anaemia	RA-related diseases	2.4	1.6–3.6	<0.01
B81	Anaemia, vitamin B12/folate deficiency	RA-related diseases	2.4	1.5–3.6	<0.01

After correction for multiple testing using -falsepositive rate control, none of these variables lost their significance.

IA, inflammatory arthritis; ICPC, International Classification of Primary Care; NA, not applicable; NOS, not otherwise specified; RA, rheumatoid arthritis.

10.1136/rmdopen-2019-001163.supp1 Supplementary data

The study was approved according to the governance code of Nivel-PCD, under number NZR-00314.045. Dutch law allows the use of EHRs for research purposes under certain conditions. According to this legislation, obtaining informed consent nor approval by a medical ethics committee are obligatory for this type of observational studies containing no directly identifiable data (Dutch Civil Law, Article 7:458).

### Statistical analysis

We first describe the presence of ICPC codes from the four predetermined groups (musculoskeletal symptoms, infections, RA-related comorbidities and chronic diseases) in the individuals with and without a diagnosis of IA. We therefore marked per quartile of the year whether a person was given an ICPC code from a particular group or not, and then summed all the cases which were coded (one or more times) into percentages of the total number of individuals that had retrospective follow-up in that quartile. Per group, based on these numbers we calculated ORs (with 95% CI, and p values) of developing IA using univariable logistic regression analysis within the time periods 6, 12 and 18 months prior to the diagnosis (or the matched end date in case of the control individuals).

Next, we performed two different approaches to predict the development of IA based on the ICPC codes within the 12-month period preceding IA. (1) *Using univariable and multivariable logistic regression analyses.* The univariable analysis was corrected for multiple testing using false-positive rate control.^19^ Because of their low individual frequency, the codes from the group of infections were combined into 11 groups (see online supplementary table 1). A backwards stepwise approach was used for the multivariable analysis, ultimately leaving only those ICPC codes with a p value <0.05. This led to one multivariable prediction model containing the ICPC codes from all groups and the diagnostic performance was described using the area under the curve (AUC) of the receiver operating curve. Age and gender were included irrespective of their significance level.

(2) *Using Classification and Regression Tree (CART) analysis*.^20^ This nonparametric statistical procedure uses hierarchical variable selection to create a decision tree, and thereby creates the best and most simple combination of variables to predict a certain outcome. In short, it examines all splitting variables (ICPC codes) and first selects the best predictor for the outcome (IA diagnosis). This process is repeated and the next steps will include the prior steps, that is, step 2 is the best predictor given the fact that the answer in the first step was taken into account, and so on. We used this approach, because it resembles the way that a GP evaluates a certain patient.

Univariable and multivariable regression analyses were performed with Stata/MP 13.0 (StataCorp, College Station, TX, USA). For CART analysis, we used SPSS version 21 (IBM Corp, Armonk, NY, USA).

## RESULTS

### Patient characteristics

**Figure 1:**
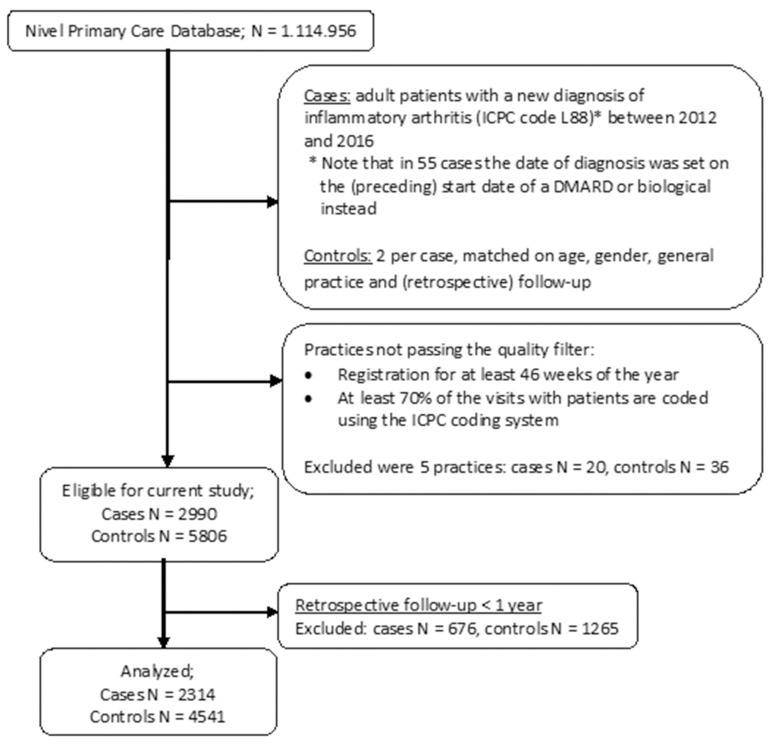
Flowchart of inclusion. DMARD, disease-modifying antirheumatic drug; ICPC, International Classification of Primary Care.

In total, 2314 IA cases with a retrospective follow-up of at least 1 year could be matched to 4541 controls (see flowchart in figure 1) from 262 practices. For 23 cases, no controls could be matched. In 53 cases, the date of diagnosis was set on the start date of the first DMARD or biological (which in all cases was the date of DMARD start, 6 cases later on used a biological), with a mean time lag of 19 months (range 1–58). In the control group, none of the included DMARDs or biologicals were used. The mean age for cases was 57.6 years (IQR 24), compared to 56.6 years (IQR 23) in the control group. Both groups contained more women than men (60%). The number of individuals with retrospective follow-up decreased further from the IA diagnosis or end point in the controls. For cases/controls, these numbers were 2314/4541, 1749/3439, 730/1430 and 172/341, respectively, at 1, 2, 4 and 6 years.

### Frequency of primary care visits prior to IA diagnosis

**Figure 2:**
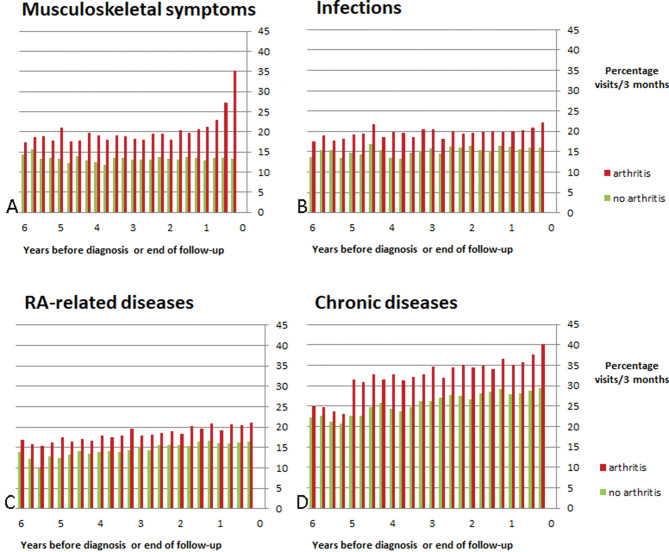
Recorded ICPC codes by the general practitioner (GP) within four groups of symptoms/diseases: (A) musculoskeletal symptoms, (B) infections, (C) inflammatory arthritis-related diseases and (D) chronic diseases. One or more visits per 3 months within a patient was counted as 1 visit, this was then divided by all patients having follow-up at that time point. ICPC, ICPC, International Classification of Primary Care.

In patients receiving the diagnosis of IA, the GP more frequently coded symptoms or diseases related to the musculoskeletal system than in control patients (figure 2). A diverging trend is already visible 4–6 years before the diagnosis, but becomes more pronounced in the final 1.5 years. Unadjusted ORs for the development of IA were 3.2 (95% CI 2.8 to 3.5, p<0.05), 2.8 (95% CI 2.5 to 3.1, p<0.01) and 2.5 (95% CI 2.2 to 2.8, p<0.01) at 6, 12 and 18 months prior to the diagnosis, respectively. The differences between cases and controls remain present over the entire study period.

Data on infections, RA-related comorbidities and chronic diseases showed a less clear pattern over time, although the higher frequency in cases than in controls seems to be present over the entire time period of 6 years. The unadjusted ORs for infections were 1.4 (95% CI 1.3 to 1.6, p<0.01), 1.5 (95% CI 1.3 to 1.6, p<0.01) and 1.5 (95% CI 1.3 to 1.7, p<0.01) at 6, 12 and 18 months, respectively. For RA-related comorbidities, these numbers were 1.3 (95% CI 1.2 to 1.5, p<0.01) for all time points, and for chronic diseases, 1.7 (95% CI 1.5 to 1.8, p<0.01), 1.7 (95% CI 1.5 to 1.9, p<0.01) and 1.7 (95% CI 1.6 to 1.9, p<0.01) at 6, 12 and 18 months, respectively.

### Individual ICPC-1 codes and their relation with IA development

Univariable logistic regression analyses showed an abundance of ICPC codes across all four groups that were statistically significantly related to the development of IA. Table 1 shows the most predominant relations (ORs ≥2.4) (for a complete overview, see online supplementary table 2). As expected from the results shown in figure 2, most of these ICPC codes came from the musculoskeletal system. The most frequent symptomatic joints were the shoulders, wrists, fingers and knees. Also, carpal tunnel syndrome was more frequently present in IA cases. Notably, specific infections were not found to be increased in future IA patients. The main associated recorded chronic diseases in future IA patients were psoriasis, inflammatory bowel disease and gout (the former two as expected due to the definition of IA).

**Table 2:** Multivariable logistic regression analysis of the relation of individual ICPC codes with IA development (N=2314 cases and N=4541 controls)

ICPC	Description	Group	OR	CI	P value	Obs*
L20	Joint symptom/complaint NOS	Musculoskeletal	7.9	5.5–11.1	<0.01	170/44
L97	Chronic internal derangement knee	Musculoskeletal	5.0	1.3–19.5	0.02	9/3
L11	Wrist symptom/complaint	Musculoskeletal	3.8	2.4–6.1	<0.01	73/30
S91	Psoriasis	Chronic diseases	3.8	2.5–5.8	<0.01	71/39
L12	Hand/finger symptom/complaint	Musculoskeletal	3.3	2.5–4.4	<0.01	179/94
D94	Chronic enteritis/ulcerative colitis	Chronic diseases	3.0	1.6–5.6	<0.01	30/17
T92	Gout	Chronic diseases	2.8	2.0–3.9	<0.01	119/69
L92	Shoulder syndrome	Musculoskeletal	2.2	1.6–2.9	<0.01	137/106
B80	Iron deficiency anaemia	RA-related diseases	2.1	1.4–2.7	<0.01	56/46
N93	Carpal tunnel syndrome	Musculoskeletal	2.0	1.3–3.0	<0.01	66/48

*Observations of number of patients (left: cases/right: controls) with that ICPC code within the last 12 months.

IA, inflammatory arthritis; ICPC, International Classification of Primary Care; NA, not applicable; NOS, not otherwise specified; RA, rheumatoid arthritis.

10.1136/rmdopen-2019-001163.supp2 Supplementary data

We then used all ICPC codes to build a multivariable prediction model for IA development using data within 12 months prior to this diagnosis. The AUC of this model was 0.69. Table 2 shows the top 10 ICPC codes (for the complete prediction model containing 32 ICPC codes and age/gender, see online supplementary table 3). The top ten includes both joint symptoms (general, wrist, hand and shoulder) as well as more specific diagnoses such as psoriasis accompanying psoriatic arthritis and chronic enteritis/ulcerative colitis accompanying ankylosing spondylitis.

10.1136/rmdopen-2019-001163.supp3 Supplementary data

### Classification and regression trees (CART)

**Figure 3:**
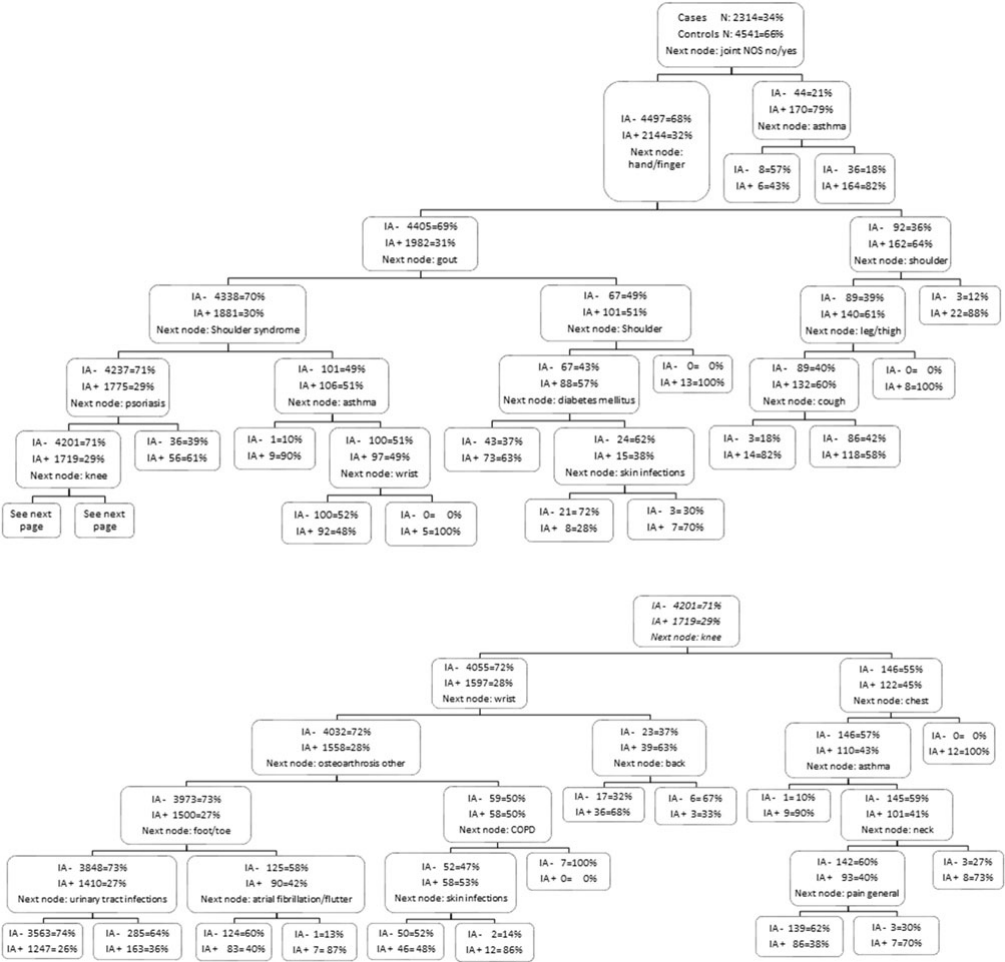
CART analysis. A CART analysis was performed to find the best and most simple combination of ICPC codes to predict IA. The a priori probability of developing IA is 34% in this dataset; thereafter, all nodes residing to the left indicate the symptom in the node above is not present and all nodes going to the right indicate the symptom is present (with coinciding percentages of developing IA). Please note that everywhere a part of the musculoskeletal system is mentioned one should read “symptom/complaint” behind it (i.e. joint NOS, hand/finger, shoulder, leg/thigh, knee, wrist, chest, back, foot/toe and neck). CART, Classification and regression tree; IA, inflammatory arthritis; ICPC, International Classification of Primary Care; NOS, not otherwise specified.

A CART analysis was performed to find the best and most simple combination of ICPC codes to predict IA. The tree is shown in figure 3. The AUC was 0.64. The classification tree starts with an a priori probability of 34% of developing IA in this dataset (predefined based on the matching process). Thereafter, all nodes residing to the right indicate the symptom mentioned in the node above is present and all nodes going to the left indicate the symptom is not present. For example, the chance of developing IA would be raised to 82% if a person has both ‘joint pain not otherwise specified’ and ‘asthma’. On the other hand, the absence of a certain variable can also lower the chance of developing IA.

## DISCUSSION

This study shows an increased frequency of musculoskeletal symptoms preceding the assumed IA-diagnosis date, mainly in the final 1.5 years. Infections, RA-related comorbidities and chronic diseases also were more prevalent in cases than in controls; however, this trend was less clear and more evenly spread out over the whole study period of 6 years. All recorded symptoms and diseases were assembled in a classification tree resembling the way a GP would detect patients to refer to the secondary healthcare system. However, the classification tree needs to be validated (AUC 64%).

The present results are in line with those of another study in which ambulatory medical care utilisation was highest in the 2 years preceding RA.^5^ As in our study, this was mainly attributed to diseases of the musculoskeletal system and connective tissue, although not further specified. We found high associations of the following symptoms/locations: knee, wrist, hand/finger, shoulder and carpal tunnel syndrome. The observation of IA starting with symptoms in hands, feet or shoulders was found before,^21^ but the present data suggest that GPs should also consider emerging IA in patients with chronic problems of the knee or carpal tunnel syndrome.

One of the early events in RA pathogenesis appears to be inflammation or infection of mucous membranes, such as in the gums, lung or gut.^22–^^28^ Rather than a one-time initiating event, the present data support a longer-term exposure, as infections as a total group were more prevalent in cases than controls during the complete follow-up. This contradicts the finding that recent infections would have a protective effect,^29^ but complements data that simultaneous development of autoimmunity and an acute phase reaction appears 4–5 years before the diagnosis of RA.^8,30,31^ Infections were combined into 11 groups, of which only genital infections, urinary tract infections, and general viral/bacterial infections were significantly related to IA in multivariable analysis (to our knowledge not linked to RA before), with low ORs of 1.4–1.5.

Comorbidities of IA have been studied extensively.^32–^^36^ Seventy percent of patients were found to have at least one chronic disease at onset of IA, which was 10% more than in control patients.^7^ We also found more RA-related comorbidities and chronic diseases in cases (ORs of 1.3–1.7). Main contributors were psoriasis, chronic enteritis/ulcerative colitis, gout, iron deficiency anaemia, vitamin B12/folate deficiency anaemia, asthma and diabetes mellitus. Gout hypothetically showed a higher association due to ICPC misclassification, as gout and IA have many similarities.^37^ To our knowledge, the other contributing factors have not been described before in the pre-disease phases, but only in the phase of established RA, psoriatic arthritis and ankylosing spondylitis.^33,36^ ^38–^^40^ On the other hand, we did not find an (expected) association with osteoarthritis^41^ and cardiovascular disease,^42^ and it thus remains unclear when the excess risk of osteoarthritis and cardiovascular disease starts.^43^

Several musculoskeletal symptoms, infections and comorbidities that were more frequently found in IA cases have not been previously described in the at-risk phase. Although for many of these variables we still have to find a scientific rationale, we have shown that with certain ICPC combinations a high percentage, up to 100%, of individuals will develop IA. This information can help GPs to earlier select individuals at higher risk for developing IA and thus aid in earlier referral. At present, the results are not robust enough to support the implementation of a prediction rule for IA in the EHRs of the GPs without further validation studies.

Our study has some limitations. First, validity of the results for the outcome IA may be lower than compared with studies in which the diagnosis of RA is supported by fulfilment of classification criteria. By definition, the present results are partly generated by patients with psoriatic arthritis or ankylosing spondylitis, the other two constituents of IA, because no individual ICPC codes exist. However, the mean age of 57 years and preponderance of females strongly suggest that the IA group mainly consisted of RA patients. Further, the diagnosis of IA is difficult for GPs to make, since it has a relatively low frequency (estimated 6 out of 400 patients with joint symptoms).^44^ This is exemplified by the fact that the IA diagnosis in a prior study has been proven to be about 71% accurate after chart review.^17^ However, this is not entirely a bad thing, since it merely reflects the GP’s way of evaluating patients. It is their job to differentiate patients that need referral to secondary care from those that do not, and all IA patients benefit from early detection. Second, besides the fact that the diagnosis of IA is difficult for GPs, possible ICPC misclassification between IA, gout and other forms of arthritis may have occurred. We cannot estimate the frequency of ICPC misclassification, as chart review is not feasible, but we know that multiple types of arthritis may coexist which makes things even more complicated.^45,46^ Also, information is lacking about other forms of arthritis, since they have not been given their own entity in the ICPC-coding. Misclassification could have led to both over- and underestimation of the found associations in the multivariable prediction model. Third, a time lag could exist between the diagnosis IA by the GP and by the rheumatologist, and in part of the IA patients, we used the first date of DMARD use as the date of diagnosis. In this large cohort, it was not feasible to perform a full chart review including free-text fields in the EHRs to correct this. Fourth, because of the limitations of our data source, no radiographic reports, autoantibody data, or personal habits such as smoking were available. Finally, the a priori chance of developing IA in this case–control study was 34%, in contrast to a prevalence of 0.5–1% for RA in the general population.^47^ Therefore, it is warranted to perform an external validation of the study results in an unselected primary care setting. In future, further classification of IA may help to unravel more details on the specific diseases that form subclassifications of the L88 ICPC code. Also, future development of the coding systems in EHRs, including for instance certain algorithms, may make diagnoses more certain and prevent a delay in recording.^17,48,49^

In conclusion, musculoskeletal symptoms, infections and comorbidities were more frequent in future IA patients than controls in the years preceding diagnosis. Primary care data, mainly on specific ICPC codes recording ‘new’ musculoskeletal symptoms such as shoulder pain, chronic pain in the knee and carpal tunnel syndrome, may help GPs to be more aware of IA development because according to our research patients are more likely to develop IA within 1.5 years. Consequently, they can consider referring these patients which may facilitate early diagnosis and treatment. Also, a higher frequency of iron deficiency anaemia, vitamin B12/folate deficiency anaemia, asthma, diabetes mellitus, genital infections, urinary tract infections and general viral/bacterial infections have not been described before to proceed the development of IA. Future validation of the ICPC codes most associated with IA development is warranted.

**Key messages**

**What is already known about this subject?**

- General practitioners (GPs) can play an essential role in earlier detection of inflammatory arthritis (IA) as they are the first professional to be consulted for health problems.

**What this study add?**

- This is the first study that gives full details of GPs electronic records in relation to the risk of developing IA.
- Musculoskeletal symptoms, infections and comorbidities were more frequent in future IA patients than controls in the years preceding diagnosis.

**How might this impact on clinical practice?**

- The use of specific ICPC codes, for example carpel tunnel syndrome, may help GPs to consider referring patients at risk for IA earlier to facilitate early diagnosis and treatment.
