# Supplementary material for: Increased primary care use for musculoskeletal symptoms, infections and comorbidities in the years before the diagnosis of inflammatory arthritis
Source: RMD Open. 2020 Jul 20;6(2):e001163. doi: 10.1136/rmdopen-2019-001163 (PMC7425115; doi:10.1136/rmdopen-2019-001163)
Supplement: Supplementary data [file rmdopen-2019-001163s001.pdf]

**Supplementary Table 1. ICPC-1 codes in four different groups**					
**Musculoskeletal symptoms/disorders**					
A01	Pain general/multiple sites	L10	Elbow symptom/complaint	L19	Muscle symptom/complaint NOS
A80	Trauma/injury NOS	L11	Wrist symptom/complaint	L20	Joint symptom/complaint NOS
L01	Neck syptom/complaint	L12	Hand/finger symptom/complaint	L29	Symptom/complaint musculoskeletal other
L02	Back symptom/complaint	L13	Hip symptom/complaint	L87	Ganglion joint/tendon
L03	Low back symptom/complaint without radiation	L14	Leg/thigh symptom/complaint	L92	Shoulder syndrome
L04	Chest symptom/complaint	L15	Knee symptom/complaint	L93	Tennis elbow
L07	Jaw symptom/complaint	L16	Ankle symptom/complaint	L97	Chronic internal derangement knee
L08	Shouder symptom/complaint	L17	Foot/toe symptom/complaint	N93	Carpal tunnel syndrome
L09	Arm symptom/complaint	L18	Muscle pain	N94	Peripheral neuritis/neuropathy
**Infections / infection-related symptoms**					
General symptoms		***Eye symptoms/diseases***		***Mouth symptoms/diseases***	
A02	Chills	F02	Red eye	D19	Teeth/gum symptom/complaint
A03	Fever	F70	Conjuctivitis infectious	D20	Mouth/tongue/lip symptom complaint
A04	Weakness/tiredness general	F72	Blepharitis/stye/chalazion	D82	Teeth/gum disease
A05	Feeling ill	F73	Eye infection/inflammation other	D83	Mouth/tongue/lip disease
A29	General symptoms/compaint other				
Viral and bacterial infections		***Ear, nose and throat symptoms/diseases***		***Skin symptoms/diseases***	
A70	Generalized tuberculosis (excluding tuberculosis lungs)	H70	Otitis externa	S10	Warts
A71	Measles	H71	Acute otitis media/myringitis	S11	Infected finger/toe
A72	Chickenpox	H72	Serous otitis media	S70	Boil/carbuncle
A73	Malaria	H73	Eustachian salpingitis	S71	Local skin infection local
A74	Rubella	H74	Chronic otitis media	S72	Scabies/other acariasis
A75	Infectious mononucleosis	R09	Sinus symptom/complaint	S73	Pediculosis/skin infestation other
A76	Viral exanthem other	R21	Throat symptom/complaint	S74	Dermatophytosis
A77	Viral disease other/NOS	R22	Tonsils symptom/complaint	S75	Moniliasis/candidiasis skin
A78	Infectious disease other/NOS	R73	Boil/abscess nose	S76	Skin infection other
A92	Toxoplasmosis	R74	Upper respiratory infection acute	S84	Impetigo
B02	Lymph gland(s) enlarged/painful	R75	Sinusitis acute/chronic	S90	Pityriasis rosea
B03	Symptoms/complaint lymph glands/immune other	R76	Tonsillitis acute	S95	Molluscum contagiosum
B70	Lympthadenitis acute	R77	Laryngitis/tracheitis acute		
B71	Lymphadenitis non-specific	R90	Hypetrophy tonsils/adenoid		
		Lung symptoms/diseases		***Gastrointestinal symptoms/diseases***	
D71	Mumps	R25	Sputum/phlegm abnormal	D22	Worms/other parasites
N72	Tetanus	R29	Respiratory symptom/complaint other	D70	Gastrointestinal infection
R71	Whooping cough	R70	Tuberculosis lungs	D72	Viral hepatitis
R72	Strep throat	R78	Acute bronchitis/bronchiolitis	D73	Gastroenteritis presumed infection
R80	Influenza (excluding pneumonia)	R81	Pneumonia	D88	Appendicitis
S70	Herpes zoster	R82	Pleurisy/pleural effusion	D95	Anal fissure/perianal abcess
S71	Herpes simplex	R83	Respiratory infection other	D98	Cholecystitis/cholelithiasis
See next page					

Other		***Genital symptoms/diseases (female and male)***			
K70	Infection of circulatory system	X70	Syphilis female	Y70	Syphilis male
L70	Infections musculoskeletal system	X71	Gonorrhoea female	Y71	Gonorrhoea male
N71	Meningitis/encephalitis	X72	Genital candidiasis female	Y72	Genital herpes male
N73	Neurological infection other	X73	Genital trichomoniasis female	Y73	Prostatits/seminal vesiculitis
Urinary tract diseases		X74	Pelvic inflammatory disease	Y74	Orchitis/epididymitis
U70	Pyelonephritis/pyelitis	X84	Vaginitis/vulvitis NOS	Y75	Balanitis
U71	Cystitis/urinary infection other	X85	Cervical disease NOS	Y76	Condylomata acuminata male
U72	Urethritis	X90	Genital herpes female		
		X91	Condylomata acuminata female		
**Rheumatoid arthritis-related comorbidities**					
A12	Allergy/allergic reaction NOS	D85	Duodenal ulcer	P17	Tabacco abuse
B80	Iron deficiency anaemia	D86	Peptic ulcer other	P19	Drug abuse
B81	Anaemia, Vitamin B12/folate deficiency	D87	Stomach function disorder	P76	Depressive disorder
B82	Anaemia other/unspecified	F71	Conjunctivitis allergic	R02	Shortness of breath/dyspnoea
D01	Abdominal pain/cramps general	K75	Acute myocardial infarction	R03	Wheezing
D03	Abdominal pain epigastric	K78	Atrial fibrillation/flutter	R05	Cough
D04	Dyspepsia/indigestion	K79	Paroxysmal tachycardia	S97	Allergic rhinitis
D06	Abdominal pain localized other	K85	Elevated blood pressure	T15	Urticaria
D08	Flatulence/gas/belching	K89	Transient cerebral ischaemia	T81	Thyroid nodule/swelling
D09	Nausia	K93	Pulmonary embolism	T83	Obesity
D11	Diarrhoea	K94	Phlebitis/thrombophlebitis	T85	Overweight
D12	Constipation	P03	Feeling depressed	Z01	Poverty/financial problems
D18	Change faeces/bowel movements	P15	Chronic alcohol abuse	Z05	Work problem
D25	Abdominal distension	P16	Acute alcohol abuse	Z06	Unemployment problem
**Chronic diseases**					
B90	HIV-infection/aids	K91	Atherosclerosis/peripheral vascular disease	R96	Asthma
D92	Diverticular disease	L89	Osteoarthrosis of hip	S91	Psoriasis (with or without arthropathy)
D94	Chronic enteritis/ulcerative colitis	L90	Osteoarthrosis of knee	T82	Goitre
K74	Ischaemic heart disease with angina	L91	Osteoarthrosis other	T86	Hypothyreoidism/myxoedema
K76	Ischaemic heart disease without angina	L95	Osteoporosis	T90	Diabetes mellitus
K77	Heart failure	N70	Poliomyelitis	T92	Gout
K86	Hypertension uncomplicated	P72	Schizophrenia	U88	Glomerulonephritis/nephrosis
K87	Hypertension complicated	R91	Chronic bronchitis/bronchiectasis		
K90	Stroke/cerebrovascular accident	R95	Chronic obstructive pulmonary disease		Note: NOS = not otherwise specified
