# Supplementary material for: Increased primary care use for musculoskeletal symptoms, infections and comorbidities in the years before the diagnosis of inflammatory arthritis
Source: RMD Open. 2020 Jul 20;6(2):e001163. doi: 10.1136/rmdopen-2019-001163 (PMC7425115; doi:10.1136/rmdopen-2019-001163)
Supplement: Supplementary data [file rmdopen-2019-001163s002.pdf]

| **Supplementary Table 2. Univariate logistic regression analysis of the relation of individual ICPC-codes with IA development** | | | | | |
|---|---|---|---|---|---|
| ICPC | Description | Group | OR | CI | p-value |
| L20 | Joint symptom/complaint NOS | Musculoskeletal | 8.1 | 5.8 - 11.3 | <0.01 |
| L97 | Chronic internal derangement knee | Musculoskeletal | 5.9 | 1.6 - 21.8 | <0.01 |
| L11 | Wrist symptom/complaint | Musculoskeletal | 4.9 | 3.2 - 7.5 | <0.01 |
| NA | Other infectious symtoms | Infections | 4.9 | 1.5 - 15.7 | <0.01 |
| L12 | Hand/finger symptom/complaint | Musculoskeletal | 4.0 | 3.1 - 5.1 | <0.01 |
| S91 | Psoriasis | Chronic disease | 3.7 | 2.5 - 5.4 | <0.01 |
| D94 | Chronic enteritis/ulcerative colitis | Chronic disease | 3.5 | 1.9 - 6.4 | <0.01 |
| T92 | Gout | Chronic disease | 3.5 | 2.6 - 4.7 | <0.01 |
| L29 | Symptom/complaint musculoskeletal other | Musculoskeletal | 2.9 | 1.9 - 4.4 | <0.01 |
| N93 | Carpal tunnel syndrome | Musculoskeletal | 2.7 | 1.9 - 4.0 | <0.01 |
| L92 | Shoulder syndrome | Musculoskeletal | 2.6 | 2.0 - 3.4 | <0.01 |
| L91 | Osteoarthrosis other | Chronic disease | 2.6 | 1.9 - 3.5 | <0.01 |
| L19 | Muscle symptom/complaint NOS | Musculoskeletal | 2.5 | 1.6 - 4.0 | <0.01 |
| B80 | Iron deficiency anaemia | RA-related diseases | 2.4 | 1.6 - 3.6 | <0.01 |
| B81 | Anaemia, Vitamin B12/folate deficiency | RA-related diseases | 2.4 | 1.5 - 3.6 | <0.01 |
| K94 | Phlebitis/thrombophlebitis | RA-related diseases | 2.3 | 1.4 - 3.8 | <0.01 |
| L08 | Shouder symptom/complaint | Musculoskeletal | 2.2 | 1.8 - 2.8 | <0.01 |
| L15 | Knee symptom/complaint | Musculoskeletal | 2.2 | 1.8 - 2.8 | <0.01 |
| L17 | Foot/toe symptom/complaint | Musculoskeletal | 2.2 | 1.8 - 2.7 | <0.01 |
| L18 | Muscle pain | Musculoskeletal | 2.2 | 1.7 - 3.0 | <0.01 |
| A01 | Pain general/multiple sites | Musculoskeletal | 2.2 | 1.4 - 3.5 | <0.01 |
| N94 | Peripheral neuritis/neuropathy | Musculoskeletal | 2.1 | 1.4 - 2.9 | <0.01 |
| T83 | Overweight | RA-related diseases | 2.1 | 1.04 - 4.3 | 0.04* |
| L16 | Ankle symptom/complaint | Musculoskeletal | 2.0 | 1.2 - 3.4 | <0.01 |
| L01 | Neck syptom/complaint | Musculoskeletal | 1.9 | 1.5 - 2.4 | <0.01 |
| L02 | Back symptom/complaint | Musculoskeletal | 1.9 | 1.5 - 2.3 | <0.01 |
| L89 | Osteoarthrosis of hip | Chronic disease | 1.9 | 1.3 - 2.7 | <0.01 |
| L09 | Arm symptom/complaint | Musculoskeletal | 1.9 | 1.2 - 3.0 | <0.01 |
| L04 | Chest symptom/complaint | Musculoskeletal | 1.8 | 1.4 - 2.3 | <0.01 |
| L13 | Hip symptom/complaint | Musculoskeletal | 1.8 | 1.3 - 2.5 | <0.01 |
| A80 | Trauma/injury NOS | Musculoskeletal | 1.8 | 1.1 - 2.7 | 0.01 |
| L90 | Osteoarthrosis of knee | Chronic disease | 1.7 | 1.3 - 2.3 | <0.01 |
| NA | Urinary tract symptoms | Infections | 1.6 | 1.3 - 1.8 | <0.01 |
| D12 | Constipation | RA-related diseases | 1.6 | 1.2 - 2.0 | <0.01 |
| NA | Viral and bacterial symptoms | Infections | 1.6 | 1.2 - 2.1 | <0.01 |
| T86 | Hypothyreoidism/myxoedema | Chronic disease | 1.6 | 1.2 - 2.0 | <0.01 |
| NA | Genital problems | Infections | 1.6 | 1.2 - 2.1 | <0.01 |
| **See next page** | | | | | |

| | | | | | |
|---|---|---|---|---|---|
| L95 | Osteoporosis | Chronic disease | 1.5 | 1.1 - 2.0 | <0.01 |
| K90 | Stroke/cerebrovascular accident | Chronic disease | 1.5 | 1.1 - 2.0 | 0.02 |
| D11 | Diarrhoea | RA-related diseases | 1.5 | 1.03 - 2.3 | 0.04* |
| NA | General symptoms | Infections | 1.4 | 1.2 - 1.7 | <0.01 |
| NA | Skin symptoms | Infections | 1.4 | 1.2 - 1.7 | <0.01 |
| R05 | Cough | RA-related diseases | 1.4 | 1.2 - 1.7 | <0.01 |
| L03 | Low back symptom/complaint | Musculoskeletal | 1.4 | 1.1 - 1.7 | <0.01 |
| NA | Lung symptoms | Infections | 1.4 | 1.1 - 1.7 | <0.01 |
| D06 | Abdominal pain localized other | RA-related diseases | 1.4 | 1.1 - 1.9 | <0.01 |
| R02 | Shortness of breath/dyspnoea | RA-related diseases | 1.4 | 1.04 - 2.0 | 0.03* |
| NA | Mouth symptoms | Infections | 1.4 | 1.02 - 1.9 | 0.03* |
| K74 | Ischaemic heart disease w. angina | Chronic disease | 1.4 | 1.01 - 1.9 | 0.04* |
| K86 | Hypertension uncomplicated | Chronic disease | 1.3 | 1.1 - 1.5 | <0.01 |
| R96 | Asthma | Chronic disease | 1.3 | 1.03 - 1.6 | 0.03* |
| K78 | Atrial fibrillation/flutter | RA-related diseases | 1.3 | 1.02 - 1.8 | 0.04* |
| K87 | Hypertension complicated | Chronic disease | 1.3 | 1.01-1.8 | 0.04* |
| L14 | Leg/thigh symptom/complaint | Musculoskeletal | 1.3 | 1.01 - 1.7 | 0.04* |
| NA | Ear, nose and throat symptoms | Infections | 1.2 | 1.08 - 1.4 | <0.01 |
| T90 | Diabetes mellitus | Chronic disease | 1.2 | 1.1 - 1.5 | <0.01 |

*These variables lost their significance after multiple testing correction using false positive rate control
Abbreviations: OR: odds ratio; CI: 95% confidence interval; NA: not applicable; NOS: not otherwise specified; IA: inflammatory arthritis
