# Supplementary material for: Increased primary care use for musculoskeletal symptoms, infections and comorbidities in the years before the diagnosis of inflammatory arthritis
Source: RMD Open. 2020 Jul 20;6(2):e001163. doi: 10.1136/rmdopen-2019-001163 (PMC7425115; doi:10.1136/rmdopen-2019-001163)
Supplement: Supplementary data [file rmdopen-2019-001163s003.pdf]

**Supplementary Table 3. Multivariate logistic regression analysis of the relation of individual ICPC-codes with IA development (N=2314 cases and N=4541 controls)**						
ICPC	Description	Group	OR	CI	p-value	Obs*
L20	Joint symptom/complaint NOS	Musculoskeletal	7.9	5.5 - 11.1	<0.01	170/44
L97	Chronic internal derangement knee	Musculoskeletal	5.0	1.3 - 19.5	0.02	9/3
L11	Wrist symptom/complaint	Musculoskeletal	3.8	2.4 - 6.1	<0.01	73/30
S91	Psoriasis	Chronic diseases	3.8	2.5 - 5.8	<0.01	71/39
L12	Hand/finger symptom/complaint	Musculoskeletal	3.3	2.5 - 4.4	<0.01	179/94
D94	Chronic enteritis/ulcerative colitis	Chronic diseases	3.0	1.6 - 5.6	<0.01	30/17
T92	Gout	Chronic diseases	2.8	2.0 - 3.9	<0.01	119/69
L92	Shoulder syndrome	Musculoskeletal	2.2	1.6 - 2.9	<0.01	137/106
B80	Iron deficiency anaemia	RA-related diseases	2.1	1.4 - 2.7	<0.01	56/46
N93	Carpal tunnel syndrome	Musculoskeletal	2.0	1.3 - 3.0	<0.01	66/48
L15	Knee symptom/complaint	Musculoskeletal	1.9	1.5 - 2.4	<0.01	177/162
L91	Osteoarthrosis other	Chronic diseases	1.9	1.4 - 2.7	<0.01	101/78
B81	Anaemia, Vitamin B12/folate deficiency	RA-related	1.9	1.2 - 3.1	0.01	45/38
L29	Symptom/complaint musculoskeletal other	Musculoskeletal	1.8	1.1 - 2.9	0.01	52/36
K94	Phlebitis/thrombophlebitis	RA-related	1.8	1.03 - 3.2	0.04	31/27
L02	Back symptom/complaint	Musculoskeletal	1.7	1.3 - 2.1	<0.01	155/169
L08	Shouder symptom/complaint	Musculoskeletal	1.7	1.3 - 2.2	<0.01	188/173
L17	Foot/toe symptom/complaint	Musculoskeletal	1.7	1.3 - 2.2	<0.01	175/163
L18	Muscle pain	Musculoskeletal	1.7	1.3 - 2.4	<0.01	102/92
L04	Chest symptom/complaint	Musculoskeletal	1.6	1.3 - 2.1	<0.01	121/136
L89	Osteoarthrosis of hip	Chronic diseases	1.6	1.1 - 2.4	0.02	56/60
N94	Peripheral neuritis/neuropathy	Musculoskeletal	1.6	1.1 - 2.3	0.02	62/60
L01	Neck syptom/complaint	Musculoskeletal	1.5	1.1 - 2.0	<0.01	116/124
NA	Genital problems	Infections	1.5	1.1 - 2.1	<0.01	88/111
L13	Hip symptom/complaint	Musculoskeletal	1.5	1.04 - 2.1	0.03	79/86
NA	Urinary tract symptoms	Infections	1.4	1.1 - 1.6	<0.01	272/359
NA	Viral and bacterial symptoms	Infections	1.4	1.05 - 1.9	0.02	99/123
T86	Hypothyreoidism/myxoedema	Chronic diseases	1.4	1.04 - 1.8	0.03	105/134
K86	Hypertension uncomplicated	Chronic diseases	1.2	1.1 - 1.4	<0.01	516/827
R05	Cough	RA-related	1.2	1.0 - 1.5	<0.01	205/286
D09	Nausia	RA-related	0.4	0.2 - 0.8	<0.01	17/50
D08	Flatulence/gas/belching	RA-related	0.06	0.01 - 0.6	0.048	1/12
NA	Age	NA	1.0	0.99 - 1.0	0.500	NA
NA	Gender	NA	0.8	0.8 - 0.9	<0.01	NA

* Observations of number of patients (left: cases/right: controls) with that ICPC-code within the last 12 months
Abbreviations: OR: odds ratio; CI: 95% confidence interval; NA: not applicable; NOS: not otherwise specified; IA: inflammatory arthritis
